# Supplementary material for: Protocol for an observational study of working conditions and musculoskeletal health in Swedish online retail warehousing from the perspective of sex/gender and place of birth
Source: PLoS One. 2024 Feb 23;19(2):e0297569. doi: 10.1371/journal.pone.0297569 (PMC10889605; doi:10.1371/journal.pone.0297569)
Supplement: Attachment — Submitted filename: Handel4.0_Protocol_Response to Reviewer_resubmitted.docx [file pone.0297569.s001.docx]

2023-11-24

Dear PLOS One Team

Thank you for the chance to revise our manuscript, PONE-D-23-06460, entitled, *Protocol for an observational study of working conditions and musculoskeletal health in Swedish online retail warehousing from the perspective of gender and race/ethnicity*.

Please find a summary of our responses to all comments. A marked copy of our revised manuscript with the changes highlighted is also included in our resubmission along with a clean copy of our revised manuscript.

The format of the submitted article meets PLOS ONE's style requirements. All funding-related text has been removed from the manuscript. We request that the following revision to the Funding Statement be made:

Current text: “KJ & SEM - Swedish Research Council for Health, Working Life and Welfare (Forte) grant number 2019-01051.  <https://forte.se/>

Desired text: “KJ & SEM - Swedish Research Council for Health, Working Life and Welfare (Forte) grant number 2019-01051

Best regards,

Jennie Jackson

Reviewer #1: Overall comments

- We have worked throughout our revised text to address your comments on sex vs gender and place of birth (PoB) vs race/ethnicity and to be very specific about which concepts were used in which analyses. Notably, we have adjusted our title and aims as follows:

**Protocol for an observational study of working conditions and musculoskeletal health in Swedish online retail warehousing from the perspective of sex/gender and place of birth.**

The specific study aims are to:

1. Describe working conditions and work-related musculoskeletal health in online retail warehousing.
2. Determine the extent to which working conditions and work-related musculoskeletal health differ between employees with respect to sex/gender and PoB in online retail warehousing.
3. Describe the extent to which sex/gender and/or PoB inequalities in working conditions and musculoskeletal health are related to organizational processes and practices in online retail warehousing.

**Additional specific changes**

Comments on frameworks and concept definitions
The authors state throughout the text that comparisons are being made and inequalities discovered relative to ‘race/ethnicity’. The definitions of race and ethnicity come on page 8, and it seems that the socially-constructed definition used here is more in line with the term ‘ethnoracialization’ or ‘racialialization’. I would suggest using those later terms instead of ‘race/ethnicity’ since it will more readily understood over those first 8 pages that the authors are not using an older definition of race which pertains to innate biological traits. This also seems like a better match with ‘gender’, which is widely understood to be a social construct and the two terms are often used together in the text.

But moreover… ethnoracialization is not under study in this paper, except through the surrogate measure of country of birth. Given that there are many ethnoracialized persons born in Sweden, country of birth will not capture the differential experience of those with, for example, darker skin or minority religion that fit in the ‘SB’ category. Although the questionnaire methods state that there are 6 demographic questions, it is not stated whether any of them address ethnoracialization (though it seems unlikely given Swedish restrictions on collecting this type of data). If ethnoracialization is not assessed in any way other than country of birth, then the contribution of the study is relative to country of birth and it should be stated clearly as such throughout (currently the first clear mention that only a surrogate measure is used appears on page 18). In the discussion or methods it could be stated that country of birth can be interpreted as a surrogate measure for ethnoracialization and/or language skills; this interpretation could be augmented/triangulated with qualitative data.

- We have made revisions throughout the text (including the title) to clearly indicate that we measured place of birth (PoB), and that we use this as a surrogate measure for a larger concept of race/ethnicity. We elected to maintain the term race/ethnicity rather than introduce ethnoracialization as we believe it will be more clear to readers what we mean, and to have continuity in terms with our previous published and highly related review entitled, A Systematic Review of Work Organization, Work Environment and Employment Conditions in Warehousing in Relation to Gender and Race/Ethnicity [1].

  To prevent any eventual confusion with race in terms of innate biological trains, we moved our definition of race and ethnicity from page 8 to the third sentence of the introduction:

*In contrast, the relationships between occupational health and gender - that is, a person’s socioculturally constructed identity as man, woman, or non-binary - or race/ethnicity - that is, social constructs related to physical traits (i.e. race) and sociocultural characteristics (i.e. ethnicity) [8] - have not yet been systematically investigated across Europe or within Sweden. (lines 45-50)*

We feel that in addition to the existing text (ex lines 152-154, 158-159) this will ensure the reader is clear on our use of the terms:

- Further, we moved the information on our use of PoB as a surrogate for race/ethnicity from page 18 to page 8, directly below the definition of race/ethnicity (and added a comment regarding language development):

*In the quantitative analyses, we use place of birth (PoB) as a surrogate measure of race/ethnicity. While PoB and cultural identity typically interact, we acknowledge that race, ethnicity, country of birth and (im)migration status and* ***language development*** *are differentiated both in theory and lived experiences (cf. [39,40]). Specifically, we accept “race is a socially constructed bodily concept, whereas ethnicity is a socially constructed cultural concept” [39], and country of birth and (im)migration status are directly measurable quantities. (line 154-157)*

Related to this, I wondered in the introduction if the cited physical size differences of 22cm are relevant to the birth countries likely to be represented among the participant group – but physical size or other physical characteristics related to country of birth do not seem to be captured in the study, and in any case would seem to undercut the later statement that it is the socially constructed categories that are of interest.

- As we do not intend to perform a height comparison in our study, we have removed the information on height differences related to place of birth from the intro. (lines 134-137)

Figure 1 is a little unclear, specifically what ‘linkages’ the orange arrows from the orange ‘inequality box’ are meant to describe. The shape is reminiscent of illustrations of confounding, but descriptions of stratified analyses in the text seem to suggest that this is more about differential relationships between risk factors on the left and health outcomes on the right. Perhaps adjustments to the figure or clarifications in the figure caption would help?

- The figure and figure legend have been updated to clarify the roll of individual worker characteristics in the model. Further, the first reference to the figure has been moved until later in the introduction so the figure heading appears in close proximity to the lines in the text where the model terms are defined. (New figure title and legend location - lines 182-184 + see revised model).

***Fig 1. – Working conditions and health in relation to sex/gender and race/ethnicity. Conceptual model outlining the linkages investigated in the current study between working conditions and health including consideration of individual worker characteristics.***

The text describes work exposure differences related to both gender (e.g. task allocation) and sex (e.g. height and body size characteristics which are not well accommodated by work stations and tools). However, figure 1 mentions only gender. Which is correct?

- We have updated figure 1 to include both sex and gender (see previous comment), since both were used in the different parts of the study.

  In the qualitative analyses, gender perspectives were examined.

In the quantitative analyses, we based our groups on self-reported data where participants were asked to rate themselves according to the Swedish words ‘kvinna’ and ‘man’ – words that are used for both woman/female and man/male, respectively. That is, there are not separate words in the Swedish language for those constructs as there are in English. However, the wording of the question was for people to report their ‘sex’. Thus in quantitative analyses, sex-based differences were assessed.

We acknowledge that a limitation of our survey question is the chance for misclassification of transgendered individuals. However, in Sweden in 2023 there were approximately 20 transgender diagnoses per 100,000 residents [2] and historically rates have been much lower. That is to say that the risk of misclassification strongly influencing our case study data descriptives is extremely low.

Methods
I encourage the authors to consider the term ‘participants’ rather than ‘subjects’. Research teams typically reside within hegemonic categories of class, citizenship, language and ethnoracialization, so reducing the agency of study participants to ‘subjects’ seems unjust and counter-productive (and in any case not in keeping with the respectful tone the paper has elsewhere).

- The section previously termed ‘Subject population’ has been revised to ‘Study population’. In all other instances, the term ‘participants’ was already in use.

P 13 consider adding ‘purposively selected’ to the description of participant selection to more clearly indicate that this is a non-probabilistic approach sample: “interviews, participants will be purposively selected from the pool of interested parties working day”

- The requested term has been added – line 264:

   *For the technical measurements and focus group interviews, participants will be* ***purposively*** *selected from the pool of interested parties working day or afternoon shifts to create the best balance between males and female*

P13 has a description of sampling to balance between ‘male’ and ‘female’. These categories are related to sex, not gender; gender equivalent terms are ‘man’ and ‘woman’. This brings up a further point: is there a plan to explicitly exclude gender-diverse categories? If not, consider stating ‘gender-balanced’ which allows for inclusion of trans- and/or non-binary persons as part of the gender story.

- See earlier comment re: man/male and woman/female terms in the Swedish language.

  In the survey question, a third category option will be given that is based on the standard Swedish survey response used at a national level, and will read ‘do not wish to categorize’.

What is the anticipated duration of the focus groups and interviews?

- The information has been added:

  *Interviews will be approximately 45-60 minutes in duration. (line 344-345)*

Note that a ‘balanced’ group in focus groups may reduce candor by reproducing existing hegemonies. What will be the approach to facilitate candid feedback from all group members?

- During the focus groups, we will keep open to such issues, listening in not only to what is said but also what is possibly left out, and ask follow-up questions where relevant. In focusing the research on inequality regimes, we do not consider power relations between participants an issue per se, as long as we can take note them and consider them in our analysis. The participating researchers will have a follow-up after each focus group, to identify and address such concerns, hence being able to take it into consideration in the formal analysis process.

Interview data analysis
The authors have provided a theoretical framework for the analysis of qualitative focus group and interview data, but have not referred to any specific qualitative approaches or methodologies. It seems that the approach is deductive tested for fit with Acker’s framework with vague allusions to iteration, but it is not clear how the text would be coded, categorized, condensed, what level of description/interpretation is anticipated. It would be helpful to the reader to state clearly the methods/methodological approaches and not just the theoretical standpoint.

- We have adjusted the text more thoroughly describe our methods (line 535-539):
  “After an inductive coding of the transcribed interviews, the first-cycle codes will be mapped to Acker’s five themes (organising practices and processes, control and compliance, shape and degree of inequality, visibility of inequality, and legitimacy of inequality) as well as to the factors Acker argues provide the most common basis for inequality (class, gender and race/ethnicity).”

Mixed methods
Quantitative comparisons and described between domestic and foreign-born participants – is it also the intention to compare quantitatively by gender?

- We will also compare quantitative data grouped by sex (self-reported – see early comments on language and questionnaire wording). The sentence has been updated to properly reflect both comparisons. (line 463-465):
   *In the quantitative analyses, we explore inequality related to sex by categorizing workers as male or female based on the self-reported data, and we will explore inequality related to race/ethnicity by categorizing workers by country of birth.*

Minor points
P7: “Even among workers perform the same tasks, the loads” should read:
“Even among workers performing the same tasks, the loads”

- The change has been made (line 129)

P8: In the statement: “Further, differential experiences of psychosocial loads between FB and domestic-born workers including lower workplace decision latitude (7,35)…” it would be helpful to the reader to state the context of the study, since the FB category can mean different things in North or South America, or Europe, or the Middle East.

- The sentence named was revised in conjunction with an earlier comment, and thus the point about FB being different in different parts of the world was no longer justified at that point in the text. Instead, the following text has been added to the introduction – lines 161-163:

  *In this study, comparisons related to PoB and race/ethnicity will specifically reflect differences in experiences between Swedish- and foreign-born workers..*

P14 Nordic Musculoskeletal Questionnaire is broader than just pain; consider listing ‘musculoskeletal symptoms’

- The change has been made (line 290)

  *The questionnaire modules consider: personal and demographic parameters (6 questions), employment conditions (4 questions), work task competence and work tasks currently performed (2 questions), workload, fatigue and recovery (12 questions – including the short SOFI questionnaire [42,43]),* ***musculoskeletal symptoms*** *(Standardised Nordic questionnaire [44], 1 question rated for 9 areas of the body parts), mental health symptoms (1 question – 4 symptoms), job satisfaction (1 question), and 4 dimensions from the Copenhagen Psychosocial Questionnaire COPSOQ III questionnaire [45], (social support from supervisor, sense of community at work, organisational justice, and work pace; in total 13 questions).*

P16 “A list of work tasks developed a priori in consult with” should read:
“A list of work tasks developed a priori in consultation with”

- The change has been made (line 328)

References

1.	Rydström K, Jackson JA, Johansson K, Mathiassen SE. A Systematic Review of Work Organization, Work Environment and Employment Conditions in Warehousing in Relation to Gender and Race/Ethnicity. Ann Work Expo Heal. 2023; wxac098. doi:10.1093/annweh/wxac098

2.	Kolk M, Tilley JL, Essen E Von, Moberg Y, Burn I. The Demography of Sweden’s Transgender Population – Patterns , Changes , and Sociodemographics. 2023; 1–40.
